# Potential of Ambient Sensor Systems for Early Detection of Health Problems in Older Adults

**DOI:** 10.3389/fcvm.2020.00110

**Published:** 2020-07-15

**Authors:** Hugo Saner, Narayan Schütz, Angela Botros, Prabitha Urwyler, Philipp Buluschek, Guillaume du Pasquier, Tobias Nef

**Affiliations:** ^1^ARTORG Center for Biomedical Engineering Research, Gerontechnology and Rehabilitation, University of Bern, Bern, Switzerland; ^2^University Clinic for Cardiology, University Hospital of Bern, Bern, Switzerland; ^3^Cardiology Clinic, IM Sechenov First Moscow State Medical University, Moscow, Russia; ^4^Neurorehabilitation Unit, Department of Neurology, University Hospital of Bern, Bern, Switzerland; ^5^DomoSafety SA, Lausanne, Switzerland

**Keywords:** ambient motion sensors, pervasive computing, preventive health information, safety, heart failure, rhythm disturbances, older adults

## Abstract

**Background:** Home monitoring sensor systems are increasingly used to monitor seniors in their apartments for detection of emergency situations. The aim of this study was to deliver a proof-of-concept for the use of multimodal sensor systems with pervasive computing technology for the detection of clinically relevant health problems over longer time periods.

**Methods:** Data were collected with a longitudinal home monitoring study in Switzerland (StrongAge Cohort Study) in a cohort of 24 old and oldest-old, community-dwelling adults over a period of 1 to 2 years. Physical activity in the apartment, toilet visits, refrigerator use, and entrance door openings were quantified using a commercially available passive infrared motion sensing system (Domosafety S.A., Switzerland). Heart rate, respiration rate, and sleep quality were recorded with the commercially available EMFIT QS bed sensor device (Emfit Ltd., Finland). Vital signs and contextual data were collected using a wearable sensor on the upper arm (Everion, Biovotion, Switzerland). Sensor data were correlated with health-related data collected from the weekly visits of the seniors by health professionals, including information about physical, psychological, cognitive, and behavior status, health problems, diseases, medication, and medical diagnoses.

**Results:** Twenty of the 24 recruited participants (age 88.9 ± 7.5 years, 79% females) completed the study; two participants had to stop their study participation because of severe health deterioration, whereas two participants died during the course of the study. A history of chronic disease was present in 12/24 seniors, including heart failure, heart rhythm disturbances, pulmonary embolism, severe insulin-dependent diabetes, and Parkinson's disease. In total, 242,232 person-hours were recorded. During the monitoring period, 963 health status records were reported and repeated clinical assessments of aging-relevant indicators and outcomes were performed. Several episodes of health deterioration, including heart failure worsening and heart rhythm disturbances, could be captured by sensor signals from different sources.

**Conclusions:** Our results indicate that monitoring of seniors with a multimodal sensor and pervasive computing system over longer time periods is feasible and well-accepted, with a great potential for detection of health deterioration. Further studies are necessary to evaluate the full range of the clinical potential of these findings.

## Introduction

One of the biggest challenges for our societies in the near future is to help the fast-growing senior population to live independently in good health and with the highest quality of life. Frailty and chronic diseases decrease the ability of elderly people to live independently. Frailty develops as a consequence of age-related decline in many physiological parameters, leading to increased risk of falls, progressive disability, need for long-term care, and increased mortality. The potential markers for the frailty syndrome are grip strength, walking speed (gait speed), clinical frailty scales, self-reported exhaustion, low physical activity, and non-intentional weight loss. These parameters can be measured by existing devices and may be used to monitor the indicators of frailty and health status. A major challenge in this regard is the correlation of motion and biological signals with events such as dizziness, falls, cardiovascular events, and progressive decline of physical and cognitive performance. An even greater challenge is the timely recognition of preventive information about health status deterioration.

Advances in technology have made pervasive computing feasible for technology-assisted independent living by embedding smart microprocessor-driven computing devices in everyday objects (for instance, appliances of smart homes) (1). There is a growing body of novel research showing that such systems are feasible and mostly accepted by seniors as well as that they can be useful for the detection of emergency situations or early changes in health status (2–4). However, evidence is still limited to few research groups and often focuses on cognitive decline specifically. We have recently been able to demonstrate the validity of physical activity measures based on simple passive infrared (PIR) sensors (5), as well as how wirelessly detected daily patterns in activities of daily living differ largely between mild cognitive impairment (MCI) subjects and age-matched controls. Other work has, for instance, shown that changes in PIR-sensor-derived motion density maps correspond to exacerbations of depression and dementia (6). Hayes et al. demonstrated how variability in PIR-sensor-derived activity and gait speed data differed between cognitively normal subjects and those with MCI (7). Urwyler et al. highlighted the difference between sensor-derived activities of daily living patterns in healthy and MCI subjects (8). Skubic et al. provided evidence on the usefulness of general health alerts that were generated on the basis of pervasive computing systems in free-living older adults (9).

In this study, we evaluate the feasibility and the acceptability of multiple pervasive computing technologies. This includes motion signals from PIR sensors, vital signs, and acceleration from a wearable sensor, as well as data from a ferroelectric-based bed sensor, throughout a period of 1 to 2 years. While a more data-driven analysis was performed by Skubic et al. (9), the aim of this study is to deliver a proof-of-concept for the use of such combined sensor systems with pervasive computing technology for the detection of clinically relevant health problems over longer time periods.

## Methods

### Study Population

The study population consists of 24 old and oldest-old seniors (mean age 88.9 ± 7.5 years, 79% females). The inclusion criteria were based on age >70 years, the ability to live in a home or apartment, and living independently. The recruitment aimed to represent a naturalistic sample of single-living, community-dwelling older adults in central Switzerland, irrespective of their cognitive status (Table 1).

**Table 1:** Participants' characteristics and demographics.

**Participant**	**Age**	**Chronic disease**	**Days recorded**			
			**DomoCare^®^**	**EMFIT QS**	**Everion^®^**	**AX3**
1	>90	Progressive dementia	292	157	76	97
2	>90	Heart failure	580	434	210	105
3	86–90	Heart failure	545	504	203	133
4	86–90		398	225	126	197
5	81–85		555	470	205	165
6	70–75		224	432	200	122
7	76–80		530	458	231	139
8	76-80		509	622	164	143
9	>90		422	303	242	139
10	>90		506	430	302	152
11	86–90	Insulin-dependent diabetes, recurrent urosepsis	529	332	227	116
12	>90	Heart failure, recurrent urosepsis	600	523	190	147
13	>90		438	352	155	172
14	>90		411	280	225	165
15	>90	Progressive dementia	353	313	158	147
16	76–80	Heart failure, recurrent tachycardia	358	215	215	158
17	86–90		357	211	152	118
18	>90	Progressive dementia, pulmonary embolism	555	380	157	168
19	>90	Recurrent pulmonary embolism, right heart failure	225	159	31	67
20	81–85	Bradycardia/pacemaker	458	395	194	134
21	>90		167	291	175	140
22	86–90		538	380	20	141
23	86–90	Insulin-dependent diabetes	194	54	0	0
24	86–90	Parkinson's disease, insulin-dependent diabetes	349	185	0	0

The study was conducted based on principles in the Declaration of Helsinki and approved by the responsible Ethics Committee of the Canton of Bern and other participating cantons in Switzerland (KEK-ID: 2016-00406). All the study participants signed and provided a written informed consent to participate in this study and permission to use their data for research and publications without restrictions.

### Ambient Sensors

Data regarding motions in the home, as well as information about entrance and fridge door openings, were recorded using the commercial DomoCare® home monitoring system for seniors (DomoSafety SA, Lausanne, Switzerland). This system included five PIR motion sensing units and two magnetic door sensors that communicate wirelessly with a base unit. The motion sensors measure motion in equipped rooms once every 2 s (0.5 Hz). The base unit collects the data and sends it to the cloud in real-time using the Global System for Mobile Communications (GSM) network. The subject's kitchen, living room, entrance, bedroom, and bathroom were each equipped with one or multiple PIR sensors (depending on the size of the room). The two door sensors were placed at the fridge and the entrance doors.

Data during sleep were recorded with EMFIT QS (Emfit Ltd., Vaajakoski, Finland) devices, which are capable of performing ballistocardiography. These sensors use thin quasi-piezoelectric films that measure even slight pressure differences as produced by the beating heart. The EMFIT QS sensors were fixed under the participants' mattresses, in proximity to the chest. The devices required no further manual intervention and transmitted data to the cloud in real-time through local WiFi and, subsequently, the GSM network. The devices extract a variety of vital signs, including heart rate, respiration rate, heart rate variability, movements in bed, sleep duration, and sleep onset delay. The proprietary heart rate and respiration rate of this sensor have recently been validated (10).

### Wearable Sensors

During the day, the participants were asked to wear an armband comprising the medical-grade Everion® (Biovotion AG, Zürich, Switzerland) sensor and an AX3 accelerometer (Axivity Ltd., Newcastle, United Kingdom). The former sensor captures a variety of vital signs, including heart rate, heart rate variability, respiration rate, galvanic skin response, and skin temperature. Its accuracy regarding heart rate and heart rate variability have been confirmed multiple times [for instance, see Barrios et al. (11)]. The latter sensor device is a state-of-the-art MEMS 3-axis accelerometer that has been well validated and used in large-scale studies like the UK Biobank Study (12). It records raw acceleration, temperature, and light. The Everion® device data were autonomously transmitted to a smartphone via Bluetooth low energy at nighttime and subsequently uploaded to the cloud. The device was charged throughout the night on an inductive charging unit, making it easy for the older adults to keep the device powered up. Data from the AX3 sensor were manually collected, and the device was charged every second week. Further details regarding the sensors can be found in Table 2.

**Table 2:** Summary of sensors used, including the sampling rates used, the sensor location, and the extracted signals.

**Sensor**	**Manufacturer**	**Sampling rate (processed)**	**Sampling rate (raw)**	**Location**	**Extracted signals**
DomoCare®	DomoSafety S.A.	-	0.5 Hz	Walls, doors	Larger motions, entrance openings/closings, fridge openings/closings
EMFIT QS	Emfit Ltd.	0.25 Hz	100 Hz	Beneath mattress	Respiration rate, heart rate, heart rate variability, large movements, bed exits, sleep stages, sleep duration, sleep onset delay, raw pressure signal (i.a.)
Everion®	Biovotion AG	1 Hz	-	Upper arm	Respiration rate, heart rate, heart rate variability, activity, interpulse interval, galvanic skin response, skin temperature (i.a.)
AX3	Axivity	-	100 Hz	Upper arm	Tri-axial acceleration, temperature, light

### Clinical Assessment

**Figure 1:**
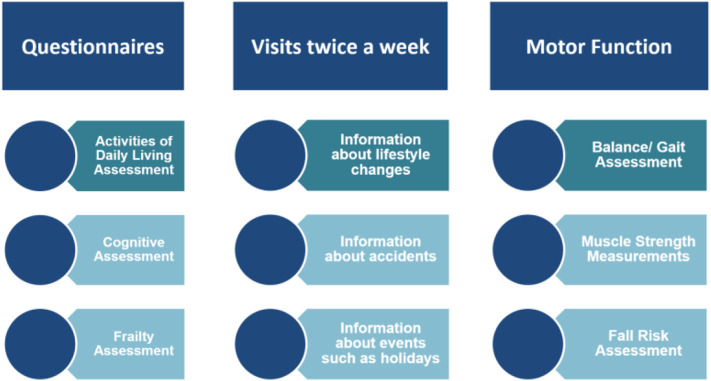
Data collection procedures for 24 old and oldest-old adults in the StrongAge Cohort Study.

The clinical assessments conducted at the beginning of the study (baseline) consisted of a battery of standardized tests targeting cognitive and motor function (Figure 1). The cognitive part included the Montreal Cognitive Assessment (MoCA) (13) and the Geriatric Depression Scale (14). The motor tests included the Tinetti Performance-Oriented Mobility Assessment (15), the Timed Up and Go (TUG) (16) test as well as muscle strength measurements for hand grip, knee extensor, and hip flexor—for all the three muscle groups, the left side and the right side strength was measured, respectively. The hand grip measurements were performed using a Jamar Plus+ Dynamometer, while knee and hip strength was assessed with a Lafayette® Manual Muscle Tester (Lafayette Instrument Company, Lafayette, IN, USA).

In addition to the baseline assessment, the muscle strength and TUG measures were assessed every 6th week, and the whole initial battery was repeated after 1 year (a different variation of the MoCA was used there to avoid memory effects).

Throughout the study duration, the subjects were visited or contacted on a weekly basis. The health-related data of the participants were obtained to verify clinical history. As part of these weekly visits, the participants were asked to fill out the EQ-5D-3L questionnaire.

### Data Analysis

After initial parsing, all collected data were stored in medical-grade, secure data centers according to Swiss regulations. To make further processing easier, the data have been initially processed to remove erroneous sensor readings. While physically impossible values have been filtered out directly (e.g., negative duration or heart rates of 0 bpm, for a living subject), less obvious but still large anomalous values were removed by means of the local outlier factor algorithm (17), which measures the local density based on nearest neighbors and allowed for the effective removal of obvious sensor artifacts.

For further analysis, the collected data were integrated into an OmniSci analytics database management system (OmniSci, San Francisco, CA, United States), allowing for efficient storage and fast query and analysis of the large amounts of collected data.

The analysis of this kind of data is complex and still an ongoing process. The sleep data shown in this work from EMFIT QS was extracted based on proprietary algorithms from Emfit Ltd. and aggregated on a daily basis for graphical display (mean aggregation for heart rate, respiration rate, and heart rate variability; sum for larger movements in bed). Activity in the apartment based on PIR motion sensors was calculated according to our previous publication (7). Transition duration was calculated based on the median time it took a participant to trigger distinct sensor pairs—thus, the time it takes to transition from one distinct room to another, a potential proxy of gait speed. Then, 14-day and 15-min moving averages have been added to make the potential trends more visible in the presented graphs.

All analyses related to this article was done using the Python programming language version 3.6 (Python Software Foundation).

### Data Privacy and Protection

The sensor data recorded in the home and the medical and behavioral information extracted from it are sensitive personal information. This mandates high technical and organizational measures for securing the data against unauthorized access. Additionally, the *perceived* data protection and privacy is at least as important. To enable a large-scale clinical usage of the aforementioned technologies, it is required to build up trust with the subjects as well as with all stakeholders regarding the protection of their data.

In the European Union, a major step in this direction has been achieved by enacting the General Data Protection Regulation, which has been in effect as a law since May 2018. Similar efforts have been undertaken in other parts of the world, notably the California Consumer Privacy Act or the Swiss data protection law—under which jurisdiction this study was collected.

The data collected in this study were handled with utmost care and attention to privacy. The data collection process was reviewed and approved by the Ethics Committee of the Canton of Bern, and collection was explicitly accepted by each participant by signing an informed consent. Technically, a privacy-by-design approach was used—storing pseudonymized data physically separately from personally identifiable data. Data access was granted to researchers based on a need-to-know rule only. To reduce the potential exposure and when technically possible, third-party cloud providers (e.g., Emfit Ltd.) only processed pseudonymized data, identified by a sensor serial number, for example. For the usage in a clinical setting, these and similar data protection approaches are implemented by the manufacturers of sensors (e.g., DomoSafety SA) and thus guarantee the privacy of the collected data.

## Results

During the study period of more than 1 year, 24 old and oldest-old participants (age 88.9 ± 7.5 years) (Table 1) were monitored by pervasive computing systems. Twelve seniors lived in their home or in an apartment. Nine of the 12 seniors living at home did not need support for daily living, whereas three of these 12 seniors had external support by home care nurses. Twelve study participants lived in a small apartment in the setting of an assisted living institution. There were 20/24 study participants who completed the study, two participants quit their study participation because of severe health deterioration and weakness, whereas two participants died during the study period.

In terms of sensor data, 242,232 person-hours were recorded with the DomoCare® system, 194,520 person-hours with the EMFIT QS, 92,592 with the Everion®, and 73,560 with the AX3. In addition, 963 status health records have been filled out throughout home visits by nursing students or nurses.

A history of chronic disease was present in 12/24 seniors: four heart failure, two symptomatic heart rhythm disturbances, two recurrent pulmonary embolism, three insulin-dependent diabetes, and one Parkinson's disease 1. The acute events during the study period included fall episodes that affected 13 seniors, seven episodes of heart failure decompensation, three episodes of urinary infection with sepsis, two cases of pulmonary embolism, three cases of pneumonia, and two cases of severe gastrointestinal infection. Episodes of heart failure decompensation were characterized by decreased activity, increased respiration and heart rate, and decreased sleep quality; heart rhythm disturbances were recognized by the bed sensor overnight. Falls were mostly preceded by increasing weakness and decreased activity.

The results from the clinical assessments are summarized in Table 3. It becomes evident that roughly four groups of participants can be distinguished according to frailty and performance. An important finding is the fact that we observed no falls in group 1, whereas there where two participants with falls in group 2, five participants with falls in group 3, and all participants with falls in group 4.

**Table 3:** Results from the clinical assessment of 24 old and oldest-old study participants.

**Variables**	**Group 1**	**Group 2**	**Group 3**	**Group 4**
Participants (*N*)	6	5	7	4
Dependence	Totally independent	Partially independent	Moderately dependent	Seriously dependent
Timed Up and Go (s)	Maximum 7.5	Maximum 9	11–29	>20
Montreal Cognitive Assessment (points)	±23	≥22	15–22	<16
Grip strength (kg)	>22	≤ 20	13–37	6–14
Medication	<5	0–6	>5	>5
Edmonton frailty scale	0–2	0–3	5–9	5–10
Mean age (years)	78	87	91	91

### Ambient Signals for Detection of Cardiopulmonary Problems

The clinical signs of heart failure include shortness of breath, rapid or irregular heartbeat, fatigue and weakness, reduced ability to exercise, swelling (edema) of legs, ankles, and feet and increased toilet visits at night (nycturia). The clinical signs of pulmonary disease include increasing respiration rate, increasing heart rate, fatigue, and weakness.

### Case Report 1

**Figure 2:**
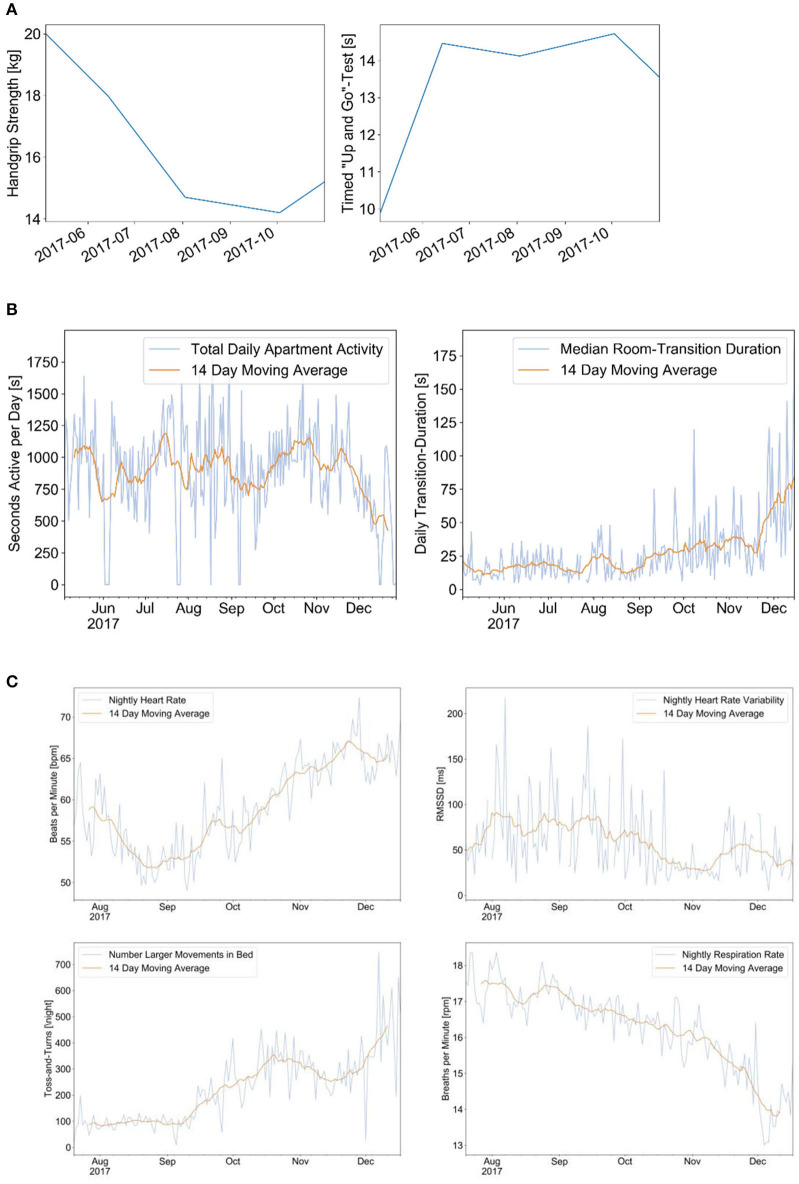
This is a case of a 97-year-oldest-old community-dwelling senior with recurrent pulmonary embolism and progressive right heart failure decompensation. The dynamics of different measurements are shown over a period of 5 months: hand grip strength and performance in the Time Up and Go (TUG) test during clinical assessments **(A)**; signals from the Emfit bed sensor indicating the heart rate, respiration rate, and motion in bed **(B)**; and signals from the passive infrared motion sensors indicating the total physical activity and the room transition time as indicator for walking speed **(C)**. **(A)** Results of hand-grip measurements (left) and TUG test (right). **(B)** Physical activity per day (left) and median room transition duration (right). **(C)** Results from Emfit bed sensor indicating the nightly heart rate (upper left), heart rate variability (upper right), number of leg movements in bed (lower left), and respiration rate (lower right).

We report an example of a 97-year-old senior with an abdominal tumor and a first episode of recurrent pulmonary embolism and right heart decompensation in June 2017. The senior decided not to go to the hospital but rather to stay home. There was a steady decrease of muscle strength as measured with a dynamometer and a decrease in performance in the Time Up and Go test starting after the acute event. The PIR motion sensors indicated a decrease of physical activity in the apartment and a slight increase of transition time as indicators of decreasing walking (gait) speed. The family doctor made a first clinical diagnosis of heart failure, not recognizing the occurrence of pulmonary embolism, and prescribed a betablocker and diuretics. From August to September, the signs of right heart failure improved, which have been indicated by a decrease of heart rate, an increase of heart rate variability, a slight decrease of respiration rate, and a relatively good sleep quality with little leg movement in bed. After a period of recovery until early December, a second episode of right heart failure decompensation occurred with progressive worsening of the clinical situation, peripheral and abdominal edema, and shortness of breath. The sensor signals indicated a further decrease of physical activity and walking speed, increasing hear rate, and restlessness in bed. The senior died at his home in December 24. The evolution of the sensor signals is shown in Figure 2.

### Ambient Signals for Rhythm Disturbances

Disturbances of the heart rhythm are common in elderly people. However, clinical experience shows that it is often difficult to correlate symptoms, such as palpitations, with heart rhythm. Clinicians use ECG registrations and, in particular, 24/48-h ECG or, in difficult cases, even implanted ECG recorders for the detection and the characterization of rhythm disturbances. The use of relatively inexpensive wearable sensors for heart rhythm analysis based on pulse wave detection or a bed sensor with ballistocardiographic capabilities to detect heart rhythm may be a suitable alternative to existing medically approved diagnostics.

### Case Report 2

**Figure 3:**
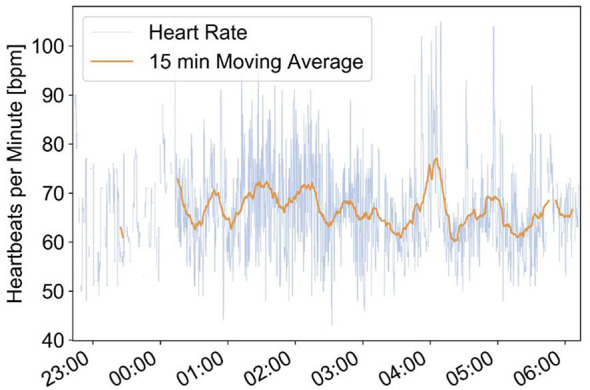
Emfit bed sensor recordings of episodes with increased heart rate between 04:00 and 05:00. This increase of heart rate corresponds well with the complaints of the senior about tachycardia with palpitations during early morning hours.

This example comes from an 86-year-old female with diabetes, arterial hypertension, and heart failure with atrial fibrillation. She had complaints about intermittent episodes of tachycardia with palpitations at night and in the early morning hours. The medication at this time included metoprolol 100 mg 1-0-0, valsartan 80 mg 1-0-0, atorvastatin 20 mg 1-0-0, metformin 500 mg ½-½-½ daily, and anticoagulation with coumadine. Episodes of significant and therefore symptomatic heart rate increases have been confirmed by the EMFIT QS bed sensor (Figure 3).

## Discussion

Using pervasive computing technology in older adults is a field of growing interest. However, few studies have been published with regard to monitoring of a naturalistic sample of independently living older adults over a longer period of time. To add evidence that the use of this technology can be feasible and useful, it is of outmost importance that more real-world evidence is being collected.

Our results indicate that monitoring of old and oldest-old community-dwelling seniors in their home or apartment with a multimodal ambient and wearable pervasive computing system over longer time periods is feasible and well-accepted. Acceptance is reflected by low attrition rates, where only two seniors dropped out because of rapid and serious health deterioration. The participants further reported to prefer sensors they do not have to interact with; this is, for instance, reflected by the difference in the number of recorded days between ambient and wearable sensors, as can be seen in Table 1. It should be noted that this difference is also due to more technical problems regarding wearable systems, such as forgetting to or having difficulties with the charging process. Regarding the AX3 sensor, we had some additional issues due to logistic problems due to manual data collection, which certainly is not ideal. Regarding the sensor types, we may thus conclude that contactless ambient sensors are the preferred approach when it comes to long-term monitoring in older community-dwelling adults. These preference for contactless technology is in line with similar research in free-living older adults (4, 18). In addition, multiple participants reported that they felt rather uncomfortable wearing something visible in public, as would happen in summer when the wearables could not be covered by long clothing. Similar stigmatization concerns have been pointed out by Peek et al. (18).

Information from sensors may be used as individual digital biomarkers. However, if taken individually, they may indicate different health problems: increased respiration rate may indicate either a pulmonary or a cardiac problem. Increased heart rate by itself is unspecific, and decreased physical activity is also an unspecific symptom. Therefore, it is evident that the combination of sensor streams to help diagnose specific health problems has great potential. The combination of decreasing activity and lower walking speed with increasing heart rate and increasing respiration rate and eventually with decreased sleep quality is a clear indicator of a serious health problem, which may develop over short or long time periods. The slow development of signs of health deterioration may allow timely preventive intervention, whereas the fast development of such signs may indicate an alarming situation or even an emergency. Further research to correlate individual sensor signals and their combinations with specific health problems and diseases is therefore an emerging task. The timely recognition of health problems could help to decrease hospitalization rates, improve the quality of life of the patients, and save costs for our healthcare system. The combined use of signals from ambient motion sensor systems and from bed sensors for heart rate and respiration rate as new digital biomarkers has the potential to be applied in wide variety of diseases: these include heart failure, atrial fibrillation, pulmonary disease, Parkinson's disease (motion and sleeping patterns), and most probably many other diseases. However, recruiting of seniors and asking them for study participation over longer periods of time have turned out to be most difficult. This is the main reason why the results from studies in larger cohorts of seniors are not available.

Nevertheless, further studies are needed to evaluate the full potential of such sensor systems.

## Limitations

One of the main limitations of PIR-sensor-derived physical activity is the fact that it can only measure in-home physical activity, which may not show the whole range of physical activities a senior engages in. Another limitation is that we cannot currently distinguish between multiple persons in the apartment; thus, the method is only applicable for seniors living alone and do not have frequent long-term visits that would significantly offset sensor readings. However, current research indicates that the problem of visit recognition may be solved in the near future. Another limitation is currently that seniors living with animals like larger dogs cannot be included because the sensor system is not yet able to distinguish motion signals between humans and larger animals.

For the Emfit bed sensor, an actual limitation is the fact that rhythm disturbances can only be detected quantitatively, not qualitatively as well, which restricts the use to the detection of bradycardia and tachycardia but does not allow precise rhythm analysis like those of ECG monitoring systems.

Whereas, single sensor systems have proven their ability to monitor vital signs and motion, the integration of different sensors in an ambient sensor system and the interpretation of combined results using pervasive computing need further evaluation under various clinical conditions.

## Conclusions

Our results indicate that monitoring of seniors with a combination of ambient sensor and pervasive computing system over longer time periods is feasible and well-accepted and that it has a great potential for detection of health deterioration. Further studies are necessary to evaluate the full range of the clinical potential of these findings.

## Data Availability Statement

The datasets generated for this study are available on request to the corresponding author.

## Ethics Statement

The studies involving human participants were reviewed and approved by the Ethics Committee of Canton Bern (KED-ID 2016-00406). The patients/participants provided their written informed consent to participate in this study and permission to use their data for research and publications without restrictions.

## Author Contributions

All authors contributed to the study design and conception and contributed to data collection and management. HS, NS, and PB have been involved in data analysis. All authors have been involved in data interpretation. HS, NS, PU, and AB have been involved in the preparation of the manuscript. All authors have reviewed and approved the manuscript.

## Conflict of Interest

PB and GP are working at Domosafety S.A., one of the manufacturers of the sensor system used. The remaining authors declare that the research was conducted in the absence of any commercial or financial relationships that could be construed as a potential conflict of interest.

## References

[1] Saha D Murkherjee A Pervasive computing: a paradigm for the 21st century. Computer. (2003) 36:25–31. 10.1109/MC.2003.1185214

[2] Lyons BE Austin D Seelye A Petersen J Yeargers J Riley T Pervasive computing technologies to continuously assess Alzheimer's disease progression and intervention efficacy. Front Aging Neurosci. (2015) 7:102 10.3389/fnagi.2015.00102 26113819 PMC4462097

[3] Rantz MJ Skubic M Miller SJ Galambos C Alexander G Keller J. Sensor technology to support aging in place. J Am Med Dir Assoc. (2013) 14:386–91. 10.1016/j.jamda.2013.02.018 23562281 PMC3683379

[4] Demiris G Hensel BK Skubic M Rantz M. Senior residents' perceived need of and preferences for “smart home” sensor technologies. Int J Technol Assess Health Care. (2008) 1:120–4. 10.1017/S0266462307080154 18218177

[5] Schütz N Saner H Rudin B Botros A Pais B Santschi V. Validity of pervasive computing based continuous physical activity assessment in community-dwelling old and oldest-old. Sci Rep. (2019) 9:9662. 10.1038/s41598-019-45733-8 31273234 PMC6609627

[6] Galambos C Skubic M Wang S Rantz M. Management of dementia and depression utilizing in- home passive sensor data. Gerontechnology. (2013) 11:457–68. 10.4017/gt.2013.11.3.004.00 24049513 PMC3773874

[7] Hayes TL Abendroth F Adami A Pavel M Zitzelberger TA Kaye JA. Unobtrusive assessment of activity patterns associated with mild cognitive impairment. Alzheimer's Dement. (2008) 4:395–405. 10.1016/j.jalz.2008.07.004 19012864 PMC2664300

[8] Urwyler P Stucki R Rampa L Müri R Mosimann UP Nef T. Cognitive impairment categorized in community-dwelling older adults with and without dementia using in-home sensors that recognise activities of daily living. Sci Rep. (2017) 7:42084. 10.1038/srep42084 28176828 PMC5296716

[9] Skubic M Guevara RD Rantz M. Automated health alerts using in-home sensor data for embedded health assessment. IEEE J Transl Eng Health Med. (2015) 3:2700111. 10.1109/JTEHM.2015.2421499 27170900 PMC4848095

[10] Ranta J Aittokoski T Tenhunen M Alasaukko-Oja M EMFIT QS heart rate and respiration rate validation. Biomed Phys Eng Express. (2019) 5:025016 10.1088/2057-1976/aafbc8

[11] Barrios L Oldrati P Santini S Lutterotti A Evaluating the accuracy of heart rate sensors based on photoplethysmography for in-the-wild analysis. In: Proceedings of the 13th EAI International Conference on Pervasive Computing Technologies for Healthcare (PervasiveHealth'19). New York, NY: Association for Computing Machinery (2019). p. 251–61.

[12] Doherty A Jackson D Hammerla N Plötz T Olivier P Granat MH. Large scale population assessment of physical activity using wrist worn accelerometers: the UK Biobank study. PLoS ONE. (2017) 12:e0169649. 10.1371/journal.pone.0169649 28146576 PMC5287488

[13] Nasreddine ZS Phillips NA Bédirian V Charbonneau S Whitehead V Collin I The montreal cognitive assessment, MoCA: a brief screening tool for mild cognitive impairment. J Am Geriatr Soc. (2005) 53:695–9. 10.1111/j.1532-5415.2005.53221.x 15817019

[14] Yesavage JA Sheikh JI. Geriatric Depression Scale (GDS): recent evidence and development of a shorter version. Clin Gerontol. (1986) 5:165–73. 10.1300/J018v05n01_0920629580

[15] Tinetti ME. Performance-oriented assessment of mobility problems in elderly patients. J Am Geriatr Soc. (1986) 34:119–26. 10.1111/j.1532-5415.1986.tb05480.x 3944402

[16] Podsiadlo D Richardson S. The timed “Up &amp; Go”: a test of basic functional mobility for frail elderly persons. J Am Geriatr Soc. (1991) 39:142–8. 10.1111/j.1532-5415.1991.tb01616.x 1991946

[17] Breunig MM Kriegel HP Ng RT Sander J. LOF: identifying density-based local outliers. In: Proceedings of the 2000 ACM SIGMOD International Conference on Management of Data. Dallas, TX (2000). p. 93–104. 10.1145/335191.335388

[18] Peek STM Aarts S Wouters EJM Can smart home technology deliver on the promise of independent living? A critical re-flection based on the perspectives of older adults. In: Handbook of Smart Homes, Health Care and Well Being. Cham: Springer (2015). p. 1–10. 10.1007/978-3-319-01904-8_41-1

